# Brain Responses before and after Intensive Second Language Learning: Proficiency Based Changes and First Language Background Effects in Adult Learners

**DOI:** 10.1371/journal.pone.0052318

**Published:** 2012-12-26

**Authors:** Erin Jacquelyn White, Fred Genesee, Karsten Steinhauer

**Affiliations:** 1 Centre for Research on Brain, Language and Music, Montreal, Canada; 2 Department of Psychology, McGill University, Montreal, Canada; 3 School of Communication Sciences and Disorders, McGill University, Montreal, Canada; University of Cambridge, United Kingdom

## Abstract

This longitudinal study tracked the neuro-cognitive changes associated with second language (L2) grammar learning in adults in order to investigate how L2 processing is shaped by a learner’s first language (L1) background and L2 proficiency. Previous studies using event-related potentials (ERPs) have argued that late L2 learners cannot elicit a P600 in response to L2 grammatical structures that do not exist in the L1 or that are different in the L1 and L2. We tested whether the neuro-cognitive processes underlying this component become available after intensive L2 instruction. Korean- and Chinese late-L2-learners of English were tested at the beginning and end of a 9-week intensive English-L2 course. ERPs were recorded while participants read English sentences containing violations of regular past tense (a grammatical structure that operates differently in Korean and does not exist in Chinese). Whereas no P600 effects were present at the start of instruction, by the end of instruction, significant P600s were observed for both L1 groups. Latency differences in the P600 exhibited by Chinese and Korean speakers may be attributed to differences in L1–L2 reading strategies. Across all participants, larger P600 effects at session 2 were associated with: 1) higher levels of behavioural performance on an online grammaticality judgment task; and 2) with correct, rather than incorrect, behavioural responses. These findings suggest that the neuro-cognitive processes underlying the P600 (e.g., “grammaticalization”) are modulated by individual levels of L2 behavioural performance and learning.

## Introduction

When adults begin learning a second language (L2), they start with an already-established first language (L1) system. Depending on the similarity between the two languages, transferring knowledge from the L1 can provide a useful basis to begin L2 communication, be it through shared phonological, lexical-semantic, or grammatical forms. It has been argued that anything that can transfer from the L1 will and that this can, in some cases, assist learning [1]. However, L1 transfer can also be problematic if the L1 and L2 systems do not map exactly onto one another, and this can lead to difficulties acquiring some aspects of the L2. What is unclear, and highly debated, is the extent to which a learner’s L1 continues to influence L2 acquisition and processing as he/she advances in L2 proficiency. Some researchers claim that late (i.e., post-puberty) L2 learners can *only* acquire grammatical structures that are present in the L1 (e.g., [2]) while others argue that qualitatively new structures in L2 can also be acquired, albeit more slowly than structures that are also instantiated in the L1 [3]. From a neuro-cognitive perspective, it has been argued that L2 acquisition in late learners is influenced by the neural networks that underpin L1 processing [1], [4]. However, it is unclear whether the L1 continues to influence (and potentially restrict) the neuro-cognitive mechanisms used for L2 processing as learners advance in proficiency. Using neuro-cognitive measures to longitudinally track the impact of learners’ L1 on L2 grammar processing is an important step towards understanding the neuro-cognitive changes that are associated with late L2 acquisition and the extent to which processing is influenced by the L1 [5].

In the present study, we report results from a 9-week longitudinal study that investigated the neuro-cognitive changes that are associated with L2 acquisition in adults participating in an intensive English-as-a-second-language course. This research sought to elucidate how learners’ L1 influences the neuro-cognitive mechanisms that underlie L2 grammar processing at progressive stages of L2 proficiency. It also sought to examine how individual differences in L2 behavioural performance are associated with different profiles of L2 neuro-cognitive processing and plasticity. Specifically, we investigated: (1) to what extent L1 background influences the neuro-cognitive basis of L2 grammar processing; (2) how L2 processing changes with L2 learning; and (3) the relationship between behavioural measures of L2 grammatical performance and L2 neuro-cognitive processing.

Many previous studies investigating age of acquisition effects on the neural bases of L2 processing have compared native speakers and L2 learners using cross-sectional designs to examine the extent to which factors such as age of L2 acquisition, L1 background, and L2 proficiency constrain L2 processing. Using event-related potentials (ERPs), this research has demonstrated that the neural basis of L2 grammar processing may be particularly sensitive to the interplay between these factors, especially at lower levels of L2 proficiency (e.g., [6]). However, the relative role of each factor is unclear. In particular, it is unclear whether late L2 learners who have attained relatively high levels of L2 proficiency can engage the same neuro-cognitive processes as native speakers for processing grammatical structures that are not used in the L1 [7], [8] or are expressed differently in the L1 and L2 [9], [10].

In native speakers, grammar processing is reliably associated with the P600 ERP component. The P600 is a positive-going wave that is typically maximal at central-parietal electrodes approximately 600 ms after the onset of the critical word in a sentence [11], [12]. The P600 has been interpreted as an index of structural reanalysis (i.e., a controlled and attention-driven process occurring during a relatively late stage in sentence processing; [13], [14]) sentence repair [11], [12], integration difficulty [15], or continued sentential analysis elicited by a mismatch between multiple levels of representation [16]. In many studies of L1 morpho-syntactic processing (e.g., subject-verb agreement), the P600 is preceded by a “left anterior negativity” (LAN) – a negative-going wave that is often maximal at left anterior electrodes between 300–500 ms after stimulus onset. The LAN has been linked to highly automatic rule-based parsing, thought to occur during early stages of morpho-syntactic analysis [17]. A biphasic LAN/P600 response has been observed in response to many classes of grammatical violations, including phrase structure (e.g., [18]) and inflectional morphology (e.g., [19]), although various other studies have reported P600 effects without a LAN (e.g., [9], [20], [21]).

In L2 learners, the presence of a P600 in response to L2 grammatical violations has been taken as evidence that they have “grammaticalized” the particular structure under investigation; that is, that they have incorporated the relevant rule-based grammatical knowledge into their online L2 processing system and engage in the same neuro-cognitive processes as native speakers when presented with a violation [22]. A LAN effect in L2 speakers has been taken as an indication that they can access and apply this knowledge automatically [6], [23]. LAN effects have also been associated with implicit, as opposed to explicit, learning experiences [24]. In contrast, the absence of these components in L2 learners has been used to suggest that processing at least certain kinds of late-acquired L2 grammatical structures may not involve the same neuro-cognitive mechanisms that underlie grammatical processing in native speakers. In particular, it has been argued that late L2 learners may be unable to exhibit native-like P600 responses when presented with L2 grammatical structures that are expressed differently in the L1 and L2 or that are not present in the L1 at all [7], [8], [9], [10]. It is thought that these L2 grammatical structures will not be salient enough to trigger the neuro-cognitive processes that are reflected by the P600 in native speakers (e.g., sentence reanalysis/repair as proposed by [17].

For example, Ojima et al. [8] compared the processing of English subject-verb agreement violations in native English speakers and late L2 learners of English who were Japanese native speakers. Because Japanese does not use grammatical morphology to encode number or person, native Japanese speakers cannot draw on relevant L1 grammatical knowledge when processing these structures in English. In response to violations, the native English speakers exhibited the aforementioned pattern of a LAN followed by a P600. This biphasic response was not observed in the L2 learners. Those with low levels of L2 proficiency did not exhibit any ERP responses, suggesting that they either did not recruit additional brain resources to process the violations or that their processing strategies varied too much to elicit a consistent ERP profile. In contrast, high proficiency L2 learners exhibited a left-lateralized negativity between 350–550 ms, as did the native speakers (i.e., a LAN); however, they did not display a P600. A lack of a P600 has also been observed in response to subject-verb agreement violations in high proficiency Chinese learners of English; Chinese grammar also does not use morphology to express number or person [7]. Together, these studies suggest that late L2 learners may be unable to exhibit a P600 in response to violations of L2 grammatical structures that they cannot transfer from their L1.

The complete absence of a P600 in these studies is striking. Previous research has documented a P600 in late L2 learners with low levels of L2 proficiency in response to morpho-syntactic violations when the structures under investigation are similar in the L1 [6], [25], [26]. The L2 learners tested by Ojima et al. [8] and Chen et al. [7], however, had high levels of English proficiency (as determined by their scores on standardized tests of English proficiency) and, overall, they performed with high accuracy on a grammaticality judgment task that was administered either concurrently with ERP testing or directly following it. Moreover, the presence of a native-like LAN without a P600 in high proficiency L2 learners is surprising given that the LAN is thought to index the recruitment of automatic morpho-syntactic processing mechanisms [17] and, thus, should be acquired at a later stage of L2 acquisition than the controlled processes reflected by the P600 [27]. To our knowledge, Ojima et al. [8] is the only study of L2 grammar processing to report a LAN in the absence of a P600.

Ojima et al. [8] suggest that the absence of a P600 in late L2 learners is a “true qualitative difference from native language processing” and that the cognitive processes reflected by the P600 “cannot be triggered by syntactic features acquired after a critical period” (p. 1223). Alternatively, the L2 learners in that study may have, in fact, displayed a P600, but it was out of the time range investigated. In both the Chen and Ojima studies (see also [10], [28]), the L2 learners’ ERP waveforms were not analysed after 1000 ms post-stimulus. It could very well be that late L2 learners who speak languages with different morpho-syntactic constraints are able to engage in the sentence reanalysis processes that are reflected by the P600, but are slower to initiate these processes and exhibit their effects only after 1000 ms. Indeed, P600s with peak latencies at around 1000 ms and later have been observed in previous studies of L2 grammar processing in low/intermediate proficiency L2 learners (e.g., [27], [29]).

P600s might also be delayed if L2 learners are required to read experimental sentences, particularly if the L1 and L2 use different writing systems that require different strategies for efficient word reading, as was the case for the Chinese and Japanese participants in the Ojima and Chen studies (for a discussion of the neural basis of reading in different languages, see [30]). Indeed, in a reading study of L2 sentence processing Steinhauer et al., [31]found that both high and low proficiency Chinese L2 learners of English exhibited a delayed P600 when reading English phrase structure violations compared to native English speakers. In contrast, native French speakers, even at relatively low levels of English (L2) proficiency, exhibited a P600 with a similar onset and peak latency as found in native English speakers. Thus, for late L2 learners, the latency of the P600 may reflect an interaction between L1 grammatical knowledge, L1 reading experiences, and L2 proficiency level. Testing for late occurring ERP responses (i.e., after 1000 ms) may help clarify whether L2 learners fail to exhibit this component when presented with L2 grammatical features that are not used in their L1 or if they are merely slower to elicit it.

Others have argued that in order to exhibit a P600, the L2 grammatical feature under investigation must not only be present in the L1, but must operate in a similar way in the two languages. Sabourin and Stowe [9] tested the processing of determiner-noun gender agreement in native Dutch speakers and two groups of Dutch L2 learners: those whose L1 was German or a Romance language (French, Italian or Spanish). While the concept of grammatical gender exists in the L1 of all participants, its expression is similar in Dutch and German and different in the Romance languages. Unlike German speakers, Romance speakers need to learn the gender of all Dutch nouns on a word-by-word basis and cannot transfer specific and surface level grammatical processing strategies from their L1. Sabourin and Stowe suggest that such transfer may be necessary for native-like sentence processing. Whereas the native Dutch speakers and the German-L2-learners-of-Dutch displayed similar P600 responses, no P600 was observed in the Romance speakers (even though the ERPs were analysed until 1500 ms). As highlighted by the authors, this lack of a P600 cannot be easily attributed to general L2 proficiency levels because both the Romance and German speakers displayed a native-like P600 in response to violations of past perfect tense, which operates in a similar manner in all three languages. Sabourin and Stowe concluded that, for late L2 learners, native-like recruitment of the mechanisms underlying the P600 may be limited to processing grammatical structures that are not only present in the L1 and L2, but expressed in a similar way in the two languages.

However, inspection of Sabourin and Stowe’s Romance speakers’ behavioural performance, compared to that of the Dutch and German native speakers, suggests an alternative interpretation. Performance on the grammaticality judgement task conducted concurrently with ERP testing was significantly higher for the native speakers and the German group than for the Romance group (who performed near chance level) in the gender agreement condition. These results contrast with those from the past tense condition where which all groups performed with high accuracy and exhibited significant P600s. The fact that the groups’ behavioural performance was significantly different in the very condition in which the languages also differ raises the possibility that the ERP results may not reflect L1 background alone, but also proficiency in the target structure. Indeed, the Romance speakers were also significantly worse than the German speakers on an offline task that required participants to identify the gender of the nouns that were used in the ERP study. This is important because knowing the gender of a noun is critical for identifying a violation of gender agreement [32] and recognizing a grammatical violation as such is necessary to elicit the P600 [20]. Perhaps the Romance speakers, despite their otherwise high levels of general L2 proficiency, had not attained sufficient knowledge of the Dutch grammatical gender system specifically in order to engage the sentence reanalysis processes that are reflected by the P600. Indeed, significant P600 effects have been reported in response to L2 gender agreement violations in learners who have no L1 experience with grammatical gender whatsoever, after they have attained high proficiency in the target language [23], [33], [34].

Viewed from this perspective, the lack of the P600 in the Romance speakers reported by Sabourin and Stowe [9] may simply reflect what the L2 learners had not yet acquired rather than what they were incapable of acquiring. When a grammatical structure does not exist in an L2 learner’s L1 (or operates differently in their L1 and L2), it may take longer to acquire compared to structures that are similar in both languages. At low levels of proficiency, L2 learners may fail to notice that it is obligatory to use the particular grammatical structure in certain cases [35] and, as a result, they will not use the same neuro-cognitive mechanisms to process it as native speakers. However, this does not preclude the possibility that L2 learning can continue to more proficient levels and that native-like neuro-cognitive processing can become realized once higher levels of proficiency have been achieved [6]. We do not yet have a clear understanding of how L1 knowledge and developing L2 knowledge interact at different stages of acquisition to shape L2 processing [5]. As highlighted by Li and Green [36] (p. 119), the field is in need of “longitudinal research into the adaptive changes triggered in response to the acquisition of a new language”. Rather than inferring developmental patterns by comparing different groups of learners who have attained high or low levels of proficiency, following a single group of learners as they acquire their L2 allows us to actually track this development directly. Moreover, examining proficiency with respect to specific grammatical structures, rather than globally, may provide a more sensitive measure of the neuro-cognitive changes that are associated with the acquisition of those structures [6].

Another important issue that is only beginning to be addressed is the extent to which individual differences in L2 proficiency and grammatical performance are associated with differences in neuro-cognitive processing profiles. A number of studies by Osterhout and colleagues [22], [25], [26], [37]–[39] demonstrate that the ERP waveforms of a group of L2 learners might not be representative of the neuro-cognitive processes available to subsets of learners who have attained either high or low levels of structure-specific proficiency. For example, Tanner et al. [26], [39] found that the amplitude of the P600 elicited in response to subject-verb agreement violations in English-learners of German correlated positively with their performance on an online grammaticality judgement task. This shows that learners who perform well behaviourally are more likely to recruit native-like processing strategies (or recruit them to a greater degree) than learners who perform poorly. In previous studies of L2 grammar processing (e.g., [9]) native-like effects may have been elicited in a subset of participants although they were masked by the use of group ERP data. This is important because null effects in a group of L2 learners have often been taken as evidence that native-like processing is unavailable to all L2 learners (e.g., [8]). Investigating the relationship between individual differences in behavioural performance with respect to specific L2 structures and the neuro-cognitive mechanisms used to process those structures may have important consequences for our understanding of whether it is possible for at least some late L2 learners to use native-like processing mechanisms.

### The Present Study

The present study had three goals: (1) to investigate whether late L2 learners can exhibit a P600 in response to violations of grammatical structures that are either absent in their L1 or that are expressed differently in their L1 and L2; (2) to track how the neuro-cognitive basis of L2 grammar processing changes as a result of participating in an intensive L2 course; and (3) to investigate the relationship between behavioural measures of L2 grammatical performance and L2 neuro-cognitive processing. To address these questions, ERPs were recorded in late L2 learners both at the beginning and the end of an intensive 9-week English-as-a-second-language course. At each ERP session, the learners read English sentences that were correct or that contained a violation of past tense regular verbs (**Table 1**). Studying learners longitudinally allowed us to directly track any neuro-cognitive changes that might be associated with the acquisition of these structures and how L1 background and L2 grammatical proficiency influence L2 processing. Moreover, studying the same learners at progressive stages of proficiency decreased some of the individual variability that is inherent to cross-sectional (between-subject) designs because each participant is compared to his/her own performance rather than to another individual.

**Table 1:** Sample Stimuli used at each Testing Session.

1a.	The teacher didn’t/did not start the lesson.
2a.	The teacher didn’t/did not started the lesson.
1b.	The teacher hadn’t/had not started the lesson.
2b.	The teacher hadn’t/had not start the lesson.

Numbers and letters refer to the four presentation lists. Half of the participants saw list 1 at session 1 and list 2 at session 2, and vice versa for the other participants. Lists a and b were counterbalanced across participants. Asterisks mark violations, critical verbs are underlined.

The L2 learners were native Mandarin Chinese and Korean speakers, allowing us to examine how late learners process L2 grammatical structures that are not present in their L1 (Chinese) or that operate differently in their L1 (Korean). In contrast to English, Chinese does not use inflectional morphology to express tense, person, or number. Thus, Chinese learners of English can rely on little L1 transfer to process English past tense; rather, the grammatical knowledge they can use to process these structures reflects what they have acquired in the L2 as adults. Korean speakers, on the other hand, can rely on some form of L1 transfer, although the situations in which they can apply their knowledge of inflectional morphology for processing our particular stimuli are different in their L1 and L2. Korean expresses simple past tense through verbal morphology (as does English); however the distinction between simple past and past perfect that was used in the present experiment does not exist in Korean (e.g., the difference between *she did not start* vs. *she had not started*). As in English, Korean can express simple past tense with negation by inflecting an auxiliary verb rather than the main verb (e.g., *she did not start* literally translates into *she start did not*); however Korean can also express the same idea by inflecting the main verb (e.g., *he no started* is also acceptable in Korean). Thus, Korean L2 learners of English need to learn that to express the simple past with negation in English, they must inflect the auxiliary verb but not the main verb (e.g., *did not start* vs. did not *started), whereas to express past perfect they must learn to inflect both the auxiliary and the main verbs (e.g., *had not started*). See **Table 1**. In other words, although Korean speakers have knowledge of inflectional morphology from their L1 to process English past tense, they need to learn when to apply this knowledge in order to accurately process the stimuli used in the current experiment.

Korean- and Chinese-L2 learners of English also differ in the nature of their L1 reading experiences, which may influence the latency of ERP effects elicited during L2 sentence reading. Like English, Korean is an alphabetic language that uses letters to encode phonemes that are assembled to form syllables and words. In both languages, word reading is thought to occur in a similar way [40]. Chinese, in contrast, is a logographic or morphosyllabic system - written characters correspond to spoken syllables, which in many cases are whole words. As a result of these writing system differences, Chinese speakers are thought to rely relatively more on orthographic processing and less on pre-lexical phonological processing during L1 reading than native English speakers [40]. Importantly, behavioural evidence suggests that when reading in their L2, Chinese L2 learners of English are slower and less accurate than Koreans who are matched in English proficiency, particularly when they are required to differentiate between words that look alike [41]. Thus, it is possible that word identification will take longer in the Chinese- compared to the Korean-speakers and that this may be reflected in P600 effects with delayed latencies. In order to observe effects that might occur with a delayed latency, we examine ERP responses until 1500 ms post-stimulus, rather than 1000 ms, as in some previous studies.

A second issue explored in this study is the extent to which differences in L2 grammatical proficiency are associated with the use of different neuro-cognitive processing mechanisms. Following Tanner et al., [26], [39] we correlated P600 effects with behavioural measures of grammatical sensitivity (i.e., the ability to differentiate well-formed and violation sentences). This extends the work of Osterhout and colleagues by investigating whether the relationship between individual differences in performance and neuro-cognitive processing that has been reported for the acquisition of grammatical structures that are similar in the L1 and L2 also holds for the acquisition of L2 grammatical structures that are either not present or realized in a different way in the L1 (see [25] for a discussion).

The sentence structures used here have been found to elicit a LAN and a P600 in native English speakers [42]. Based on previous work with low/intermediate proficiency L2 learners (e.g., [6], [8], [27]), we did not expect the Chinese or Korean participants to exhibit a LAN. As noted earlier, the LAN is thought to reflect implicit rule-based processing that is automatically triggered in response to a violation of morpho-syntax and, thus, is usually associated with near-native levels of L2 proficiency. Thus, it is likely that these processes will become available only after years of L2 exposure, and not after 9 weeks of instruction [6]. Therefore, our focus of interest was on whether the L2 learners in the present study would exhibit proficiency-related changes in the P600 component, which would suggest the “grammaticalization” of L2 morpho-syntax [22] and the recruitment of sentence reanalysis processes that are used by native speakers during morpho-syntactic processing [17].

Using different theoretical frameworks one could make different predictions as to whether the Chinese or Korean participants would exhibit P600 effects. Following the claim that L2 learners cannot exhibit P600s in response to L2 grammatical structures that are not instantiated in the L1 [7], [8], one would expect no P600 for the Chinese speakers; although P600s may be observed for the Korean speakers, as they could rely on at least some L1 transfer. In contrast, Tokowicz and MacWhinney [10] have argued that native-like processing is unavailable for L2 grammatical structures that are *different* from those in the L1, but may be possible for structures that are absent from the L1. This is because when the L1 and L2 provide conflicting interpretations of a given grammatical structure, the stronger L1 interpretation will prevail. This on-line competition between the two languages is thought to continue to influence L2 processing even at higher levels of L2 proficiency. Thus, these authors would predict no P600 for the Koreans, whereas the Chinese speakers may exhibit P600s by the end of the L2 course. Finally, Sabourin and Stowe [9] propose that L2 learners will exhibit a P600 only when they can transfer surface-level similarities between their two languages. In this case, we would expect to see no P600 for either group at either testing session.

Alternatively, if (as proposed in [6]) it is learners’ L2 proficiency level that is an important predictor of neuro-cognitive processing patterns, then we would expect to see P600s at session 2 for both groups, if the learners succeed at “grammaticalizing” the target structures (i.e., incorporate the relevant grammatical knowledge into their online language processing system; [22]). Moreover, P600 amplitudes should correspond to behavioural performance, as measured by grammatical sensitivity. If the Korean and Chinese speakers display P600 effects after intensive L2 instruction, it would provide evidence against the notion that the L1 grammatical system continues to limit L2 neuro-cognitive processing once intermediate levels of L2 proficiency have been attained. By examining ERP responses as a function of L1 background and L2 grammatical performance both before and after participating in an intensive L2 course, we were able to investigate how learners’ L1-background and their level of L2 proficiency modulated learning-induced changes in L2 processing at early and later stages of proficiency.

## Methods

### Ethics Statement

This research conforms to APA standards for ethical treatment of participants and has received approval from McGill University’s Research Ethics Board. Written informed consent was obtained and the rights of the participants were protected.

### Participants

Thirty-two late L2 learners of English participated in this study. Sixteen spoke Korean as an L1 (20–28 years old, *M* = 22.6, 13 female) and 16 spoke Mandarin Chinese as an L1 (18–38 years old, *M* = 23.9, 7 female). There was no age difference between the Korean and Chinese participants at the time of testing [*t*(30) = 0.77, *p*>.20]. An additional 9 participants (5 Korean) were tested but excluded from the analyses because of excessive movement, eye-blink or alpha wave artifacts contaminating the EEG signal (in at least one of the sentence conditions during one of the testing sessions; see ERP recording and analysis section), and 3 were excluded because they did not return for the second testing session. Participants gave written informed consent, were paid for their participation, had normal or corrected-to-normal vision, and reported no history of hearing, language, speech or neurological disorders. All were right-handed (assessed using self-report and the Edinburgh handedness inventory; [43]) and reported comparable educational backgrounds (i.e., most were currently undergraduate students or had recently graduated). Two cohorts of participants were recruited over two consecutive summer language programs in order to increase the sample size. Recruitment of additional participants was not possible because it ran the risk of introducing confounds due to significant changes in the course itself (e.g., course materials, content, instructors etc.). All participants were foreign students living temporarily in Canada for the purpose of studying English in an intensive 9-week English-as-a-second language course at McGill University. They were enrolled in an intermediate level class (as determined by the school’s placement test). The course provided intensive training on a range of English language skills (e.g., grammar, pronunciation, vocabulary). The goal of the course was to prepare non-native speakers for full time study in an English language university in which academic style writing and oral presentation skills would be required of them. Classes were composed of students from a variety of L1 backgrounds. As a result, instruction took a “one size fits all” approach that provided little explicit information about how L2 grammatical structures and word-level reading strategies may differ from those in their L1.

At the first testing session, we administered a cloze test of English proficiency that has been used as a general indicator of L2 proficiency in previous studies (e.g., [44]). The test consisted of a one-page passage with approximately every seventh word missing, 30 in total. They were required to read the text and fill in the missing words by selecting a word from among 4 multiple-choice options. On average, both groups performed rather poorly on the test, indicating similar low levels of general L2 proficiency at the start of the study. There was no significant difference between the Korean (*M* = 45.7%; *S.D.* = 17.1) and Chinese (*M* = 51.9%; *S.D.* = 18.3) participants [*t*(30) = 0.98, *p*>.20]. As for their final marks in the English course at the end of the study, no significant difference was observed between the Korean (*M* = 67.9%; *S.D.* = 8.8) and Chinese (*M* = 69.1%; *S.D.* = 10.4) groups either [*t*(28) = 0.36, *p*>.20], indicating similar levels of L2 proficiency at the second testing session as well (**Table 2**).

**Table 2:** Participant Information.

	Koreans		Chinese	
	Session 1	Session 2	Session 1	Session 2
Cloze test of English proficiency (%)	45.7 (17.1)	–	51.9 (18.3)	–
Final mark in course (%)	–	67.9 (8.8)	–	69.1 (10.4)
L2 self-rating test (7 point scale)				
- Listening	3.8 (1.0)	3.9 (0.9)	3.5 (1.0)	4.3 (1.1)
- Reading	4.0 (1.1)	4.2 (1.0)	4.1 (1.0)	4.6 (0.7)
- Pronunciation	3.7 (1.2)	3.8 (0.9)	4.2 (1.4)	4.4 (0.7)
- Fluency	3.6 (1.2)	3.5 (1.0)	3.8 (1.0)	4.3 (0.7)
- Vocabulary	3.6 (1.0)	4.0 (0.9)	3.8 (1.0)	4.3 (0.8)
- Grammar	3.9 (0.9)	4.4 (1.1)	4.4 (1.0)	5.1 (0.0)
- Total *	3.8 (0.8)	4.0 (0.8)	4.0 (0.8)	4.5 (0.4)
Daily use of English as % of total language use	66.2 (22.7)	67.3 (13.5)	59.7 (25.7)	66.3 (20.2)

* Session 2> Session 1 *p*≤.005.

At each session, participants self-rated their abilities in English on 6 dimensions (listening, reading, pronunciation, fluency, vocabulary and grammar) using a 7-point scale (1 = no proficiency at all, 7 = like a native speaker; **Table 2**). Potential L1 or session differences were analyzed with a repeated measures ANOVA with the 6 dimensions and session as within-subjects variables and L1 as a between-subjects variable. Overall, participants rated their English abilities higher at session 2 (*M* = 4.2) than session 1 [*M = *3.9; Sess: *F*(1,30) = 9.07, *p*≤.005]. No significant main effect or interactions with L1 were observed, indicating that the Korean and Chinese participants perceived their own English abilities as similar.

The participants completed language background questionnaires that provided information about their previous and current English experiences. Previous English exposure was assessed by asking participants to report how much English they used at home and at school (as a percentage of total language use) between the ages of 0–4, 5–11, 12–14, 15–16, 17–18 and 19+. Neither the Koreans nor the Chinese reported substantial exposure to English before the age of 12. Thus, according to Birdsong [45] both groups can be classified as late L2 learners. To test whether the Korean and Chinese groups differed in lifetime (and in particular childhood) English exposure, a repeated measures ANOVA was run using L1 group as a between-subjects variable; age (0–4, 5–11, 12–14, 15–16, 17–18, 19+) and location (home, school) were within-subjects variables. The Greenhouse-Geisser [46] correction was applied to analyses involving the age factor (as it involves more than one degree of freedom). This revealed no significant main effect or interaction with the factor L1 group, indicating no significant difference in the amount of English exposure that the Korean and Chinese groups reported receiving as children and adolescents. As seen in **Table 3**, both groups reported limited English use throughout their lives, particularly as children.

**Table 3:** Lifetime English Exposure.

	Koreans		Chinese	
Age (years)	Home	School	Home	School
0–4	0.1 (0.3)	1.3 (3.4)	0 (0)	0 (0)
5–11	2.3 (4.4)	4.1 (7.4)	0.6 (2.5)	3.3 (5.7)
12–14	5.1 (8.9)	14.9 (14.0)	0.6 (2.5)	8.6 (7.8)
15–16	3.2 (6.0)	22.7 (14.2)	0.6 (2.5)	11.5 (11.7)
17–18	3.6 (5.9)	24.8 (16.4)	1.6 (5.1)	19.6 (25.6)
19+	6.6 (10.7)	26.9 (22.4)	15.9 (31.7)	36.7 (37.6)

Average English use (as a percentage of daily total language use) throughout childhood and adolescence. Means are reported with standard deviation in parenthesis.

Current English exposure was assessed at both testing sessions by asking participants to report their current use of English and their L1 (as a percentage of their total daily language use within the week of the testing session; **Table 2**). This was analyzed using a repeated-measures ANOVA with session as a within-subjects variable and L1 as a between-subjects variable. This analysis revealed no main effect or interaction involving L1 or session (*ps* >.10), indicating similar English use by both groups at both sessions.

### Stimuli

At each session, participants read 72 experimental sentences (36 correct, 36 containing a violation) that tested their processing of the inflection rules governing the past tense of regular verbs in English. The stimuli were simple active-voice sentences consisting of 5–9 high frequency words. They were based on stimuli used in previous studies with English native speakers [42], with the vocabulary adapted for low proficiency L2 learners of English. See **Table 1** for examples of the sentences (asterisks mark violations, critical verbs are underlined). These sentences were randomized among 152 filler sentences containing other types of morpho-syntactic anomalies (subject-verb agreement and phrase structure), which will be described in another paper.

Sentences were designed to avoid ERP artifacts that can arise when the critical word and preceding baseline interval differ between the correct and violation sentences. Thus, in both conditions, 4 versions of each test sentence were created to ensure a balanced experimental design: the correct and violation contrast involved the identical verb form and preceding sentence context (see [47] for more discussion of stimulus design issues). Half of the sentences were grammatically correct and half contained a violation of English past tense (simple past or past perfect) involving a regular verb. The correct versus violation contrasts were created by manipulating the pre-target auxiliary verb, allowing us to compare ERP responses to correct and violation sentences involving identical verb forms. Half of the critical verbs used bare stem forms (e.g., *didn’t start* and *hadn’t start) and half used *–ed* suffixed participles (e.g., *hadn’t started* and *didn’t started); half were preceded by the auxiliary *do* and half by the auxiliary *have.* All of the items were negated since negation was needed to license *do.* In order to vary the position of the critical verb in the sentence, half of the items contained the contracted form of the auxiliary and negation (didn’t/hadn’t) and half contained full forms (did not/had not); in half the subject was a pronoun (he/she), in half it was a lexical noun phrase (e.g., the customer).

Participants were presented with different lists of sentences at each testing session. To create the lists, we first developed 72 sentences (each containing a different critical verb). Four versions of each sentence were then created according to the manipulations described above (see **Table 1**) and were evenly assigned to the four presentation lists (1A, 1B, 2A, 2B). No verb was repeated in a given list. Participants saw different forms of the critical verbs at each testing session; if a given verb was presented with inflection at the first session (e.g., *hadn’t started*), it was presented without inflection at the second session (e.g., *didn’t start*). The sentences were also counter-balanced across A and B lists so that a given verb form was presented in a correct sentence in one list and as a violation in the other (e.g., *didn’t start* vs. * *didn’t started*). Half of the participants were presented with a “1” list at the first session and a “2” list at the second session (e.g., 1A and 2A) and, vice versa, for the remaining participants. As a result of this procedure, when the ERPs were averaged across participants, the same critical word and preceding context appeared in both correct and violation sentences. This design ensures that ERP effects are a result of the violation per se and not confounded with lexical differences between the critical words or the contexts preceding the target word (see [47] for a discussion of baseline problems in many other studies).

### Procedure

Participants were tested twice: once after the first week of the intensive English course and then during the last two weeks of the course. At each testing session, they were seated comfortably in a sound-attenuated room, approximately 70 cm in front of a computer screen that displayed the stimuli. They were given specific instructions in English (both verbal and written) about the task and were asked not to blink or move while the stimuli were being presented. They were instructed to read each sentence carefully and to judge it for grammatical correctness by pressing one of two mouse buttons in response to a visual prompt at the end of each trial. The experiment began with the presentation of 8 practice sentences, followed by a short break in which they could ask questions. Each test trial began with the presentation of a fixation cross (500 ms) in the centre of the screen followed by sentences that were presented word-by-word in the centre of the screen (300 ms per word at an inter-stimulus interval of 200 ms). The response prompt (“*good?*”) was presented 1000 ms after offset of the last word and remained on the screen until the participants responded with a button press or 5 seconds had elapsed. After a subsequent ‘eye blinking’ interval of 1500 ms, the next trial began. Prior to the ERP session, participants completed the language-background questionnaires. Each testing session lasted for 2.5–3.0 hours, including short breaks.

### ERP Recordings and Analysis

Continuous EEG was recorded from 19 cap-mounted tin electrodes according to the international 10–20 system and digitized online at 500 Hz. Recordings were referenced to the left ear lobe and re-referenced off-line to averaged left−/right-mastoids. Eye movements were monitored by additional electrodes placed at the outer canthus of each eye (EOGH) and above and below the left eye (EOGV). Electrode impedances were kept below 5 kΩ. For approximately half of the participants, Compumedics/NeuroScan NuAmps amplifiers were used to amplify the EEG and EOG signals at the first session, whereas for the remaining participants and sessions Compumedics/NeuroScan SynAmps2 amplifiers were used. As no difference was found in the data obtained from the two amplification systems, they were collapsed together for subsequent analyses.

Offline, the EEG was filtered with a phase-true 0.3–30 Hz band-pass filter using the EEProbe software package (Advanced Neuro-Technology, ANT; Enschede, the Netherlands). Data were screened for eye movements, muscle, and other noise artifacts. Participants were included in further analyses if they contributed a minimum of 20 artifact-free trials for the correct and violation sentences at each session. On average, participants contributed 75% artifact-free trials (range: 56%–100%). A repeated measures ANOVA using Session (1 or 2) and Condition (correct or violation sentences) as within-subjects variables and L1 as a between-subjects variable revealed no significant difference in the number of trials that each L1 group contributed at each session and for each condition (*p*s >.10). After pre-processing the data, artifact-free ERP responses were averaged for each participant for each condition (i.e., correct and violation sentences) and testing session. This was done for a 1600 ms interval, time-locked to the onset of the critical verb, including a 100 ms pre-stimulus baseline interval.

Single-subject ERP averages can be based on trials that correspond to correct behavioural responses only (i.e., “response contingent” analyses) or on “all trials” irrespective of behavioural accuracy. Each approach has advantages and disadvantages. For example, it is possible that participants exhibit larger P600 effects in response to violation sentences that they deem unacceptable (i.e., correct rejections) compared to violation sentences that they perceive as correct (i.e., misses) [20]. Let us assume this is the case and assume that, for the present study, behavioural performance may also improve between sessions. Under this scenario, it would be unclear from the analysis of “all trials” whether any change in ERP effects reflects a *quantitative* change in neuro-cognitive processing (due to a larger proportion of trials with correct responses at session 2 versus session 1) or a *qualitative* change in how trials corresponding to correct responses were processed (i.e., P600 effects emerged at session 2 that were not present at session 1 whatsoever). By analyzing the ERP effects corresponding to “correct only trials”, we can distinguish between these possibilities. If significant P600 effects at session 2 are observed that are not present at session 1 when only correctly-answered trials are entered into the analyses, then it would suggest a true qualitative change in processing and the recruitment of neuro-cognitive processes that were not available to the L2 learners at session 1. In this respect, the analysis of “correct only trials” is advantageous.

On the other hand, significant changes in ERP components have been observed in L2 participants even before corresponding changes in behavioural measures of language processing occurred, suggesting that ERP measures may be more sensitive to learning progress than behavioral measures [48], [49]. Consequently, trials that correspond to incorrect behavioural responses may nevertheless elicit ERP effects in L2 learners. By discarding ERP trails based on behavioural responses, we may lose valuable information about the neuro-cognitive changes that co-occur with increasing L2 proficiency. Response-contingent analyses might also result in the exclusion of participants with an inadequate number of correctly answered trials at both testing sessions – an issue that is particularly problematic for longitudinal research with low proficiency participants. In this respect, analysis of “all trials” (i.e., trials corresponding to both correct and incorrect behavioural responses) would be advantageous.

Most ERP studies of L2 grammar processing have conducted either response-contingent analyses (e.g., [23], [33], [50]) or analyses of all trials (e.g., [8], [10], [28], [39]). In the present study, we conducted both types of analyses in an effort to better understand whether any change in ERP effects we may observe between sessions reflects a quantitative or qualitative change in neuro-cognitive processing. For the response contingent analyses, participants were included in the analysis if they contributed at least 12 correctly-answered artifact-free trials for each condition (i.e., 1/3 of total sentences). This resulted in the exclusion of 11 participants (6 Koreans). The 21 remaining participants who were included in these analyses contributed, on average, 22 trials for each condition and session.

For both sets of analyses, the mean amplitude of ERP waves was analyzed within two time windows (early: 500–700 ms and late: 750–950) based on previous studies of L2 P600 effects (e.g., [9], [10], [28], [29] and visual inspection of the grand averages for each L1 group. For each time window, repeated-measures ANOVAs were performed on 12 lateral (F3, F4, C3, C4, P3, P4, F7, F8, T3, T4, T5, T6) and 3 midline (Fz, Cz, Pz) electrodes. For the lateral electrodes, L1 (Chinese, Korean) was a between-subjects factor and the within-subjects factors were: Condition (correct or violation), Session (1 or 2), Hemisphere (left or right), Ant-Post (anterior, central, parietal), and Laterality (lateral-lateral, medial). For the midline sites, the factors were: L1 (Chinese, Korean), Condition (correction or violation), Session (1 or 2) and Ant-Post (anterior, central, parietal). Results are reported for main effects and interactions that involve at least one condition factor. The results of the midline analyses are reported only when they yielded results that were not revealed in the analyses of the lateral electrodes. The Greenhouse-Geisser correction was applied to all analyses involving the Ant-Post factor (as it involves more than one degree of freedom) and corrected *p* values are reported.

### Analysis of Behavioural Data

Following Tanner et al. [39] and Morgan-Short et al. [24] behavioural results (i.e., grammaticality judgments obtained in the EEG experiment) were quantified using d-prime scores [51]. D-prime scores provide an unbiased measure of grammatical sensitivity – participants’ ability to discriminate the correct and violation sentences. Scores were calculated based on performance on the grammaticality judgment task for each participant at each session using the following formula: *d’* = *Z*(hit rate)−*Z*(false alarm rate). These scores were analyzed using a repeated measures ANOVA with Session (1, 2) as a within-subjects factor and L1 (Korean, Chinese) as a between-subjects factor.

## Results

### Behavioural Results

Mean grammatical sensitivity (d-prime) scores for the L1 groups at each session are presented in **Table 4** **.** Overall, grammatical sensitivity improved substantially from session 1 to session 2 [Sess: *F*(1,30) = 20.04, *p*<.001]. No effects or interactions with L1 background were observed (*p*s >.10), indicating no significant difference in the Chinese and Korean participants’ performance at either sessions or in the improvement they experienced throughout the duration of the course. For reference, mean accuracy for the correct and violation sentences at both sessions are also presented in **Table 5** **.**

**Table 4:** Grammatical sensitivity (d-prime) scores at each session for the Korean and Chinese participants.

	Session 1	Session 2	Overall
**Korean**	1.41 (1.12)	2.15 (1.32)	1.78 (1.26)
**Chinese**	1.27 (0.99)	1.96 (1.11)	1.62 (1.09)
**Overall**	1.34 (1.04)	2.06 (1.20)	1.70 (1.17)

Mean values are reported with standard deviations in parentheses.

Note that a complete inability to discriminate (i.e., chance level performance) would yield a d-prime score of 0 and that d-prime scores above 2.5 correspond to very high levels of sensitivity (i.e., proportion correct over 0.90; Macmillan & Creelman, 2005).

**Table 5:** Mean accuracy for the correct and violation sentences at each session for the Korean and Chinese participants.

	Session 1			Session 2		
	Correct	Violation	Overall	Correct	Violation	Overall
**Korean**	84.2 (12.3)	58.2 (23.0)	71.2 (15.4)	87.8 (11.1)	72.9 (27.2)	80.4 (17.0)
**Chinese**	76.6 (18.4)	63.7 (18.5)	70.1 (13.8)	81.9 (13.1)	76.9 (15.8)	79.4 (12.9)
**Overall**	80.4 (15.9)	60.9 (20.7)	70.7 (14.4)	84.9 (12.4)	74.9 (22.0)	79.9 (14.8)

Mean values (percent correct) are reported with standard deviations in parentheses.

### ERP Results: Analysis by L1 Groups (All Trials)

**Figure 1:**
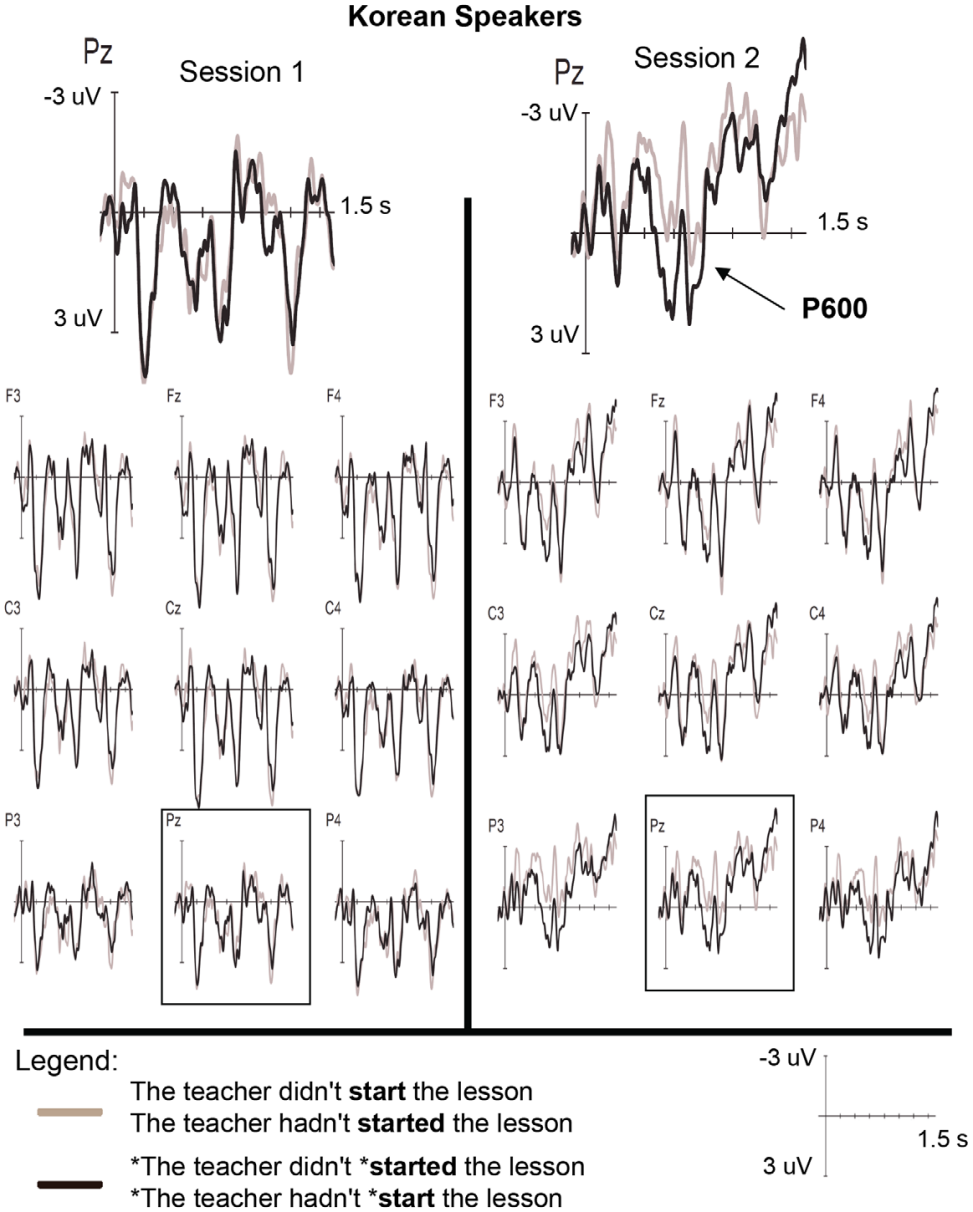
ERP data for Korean participants. Averaged ERPs for the Korean participants at session 1 and 2 for analysis of all trials. All time specifications are relative to the onset of the critical word. The Koreans exhibited a significant P600 at session 2 that was not present at session 1.

**Figure 2:**
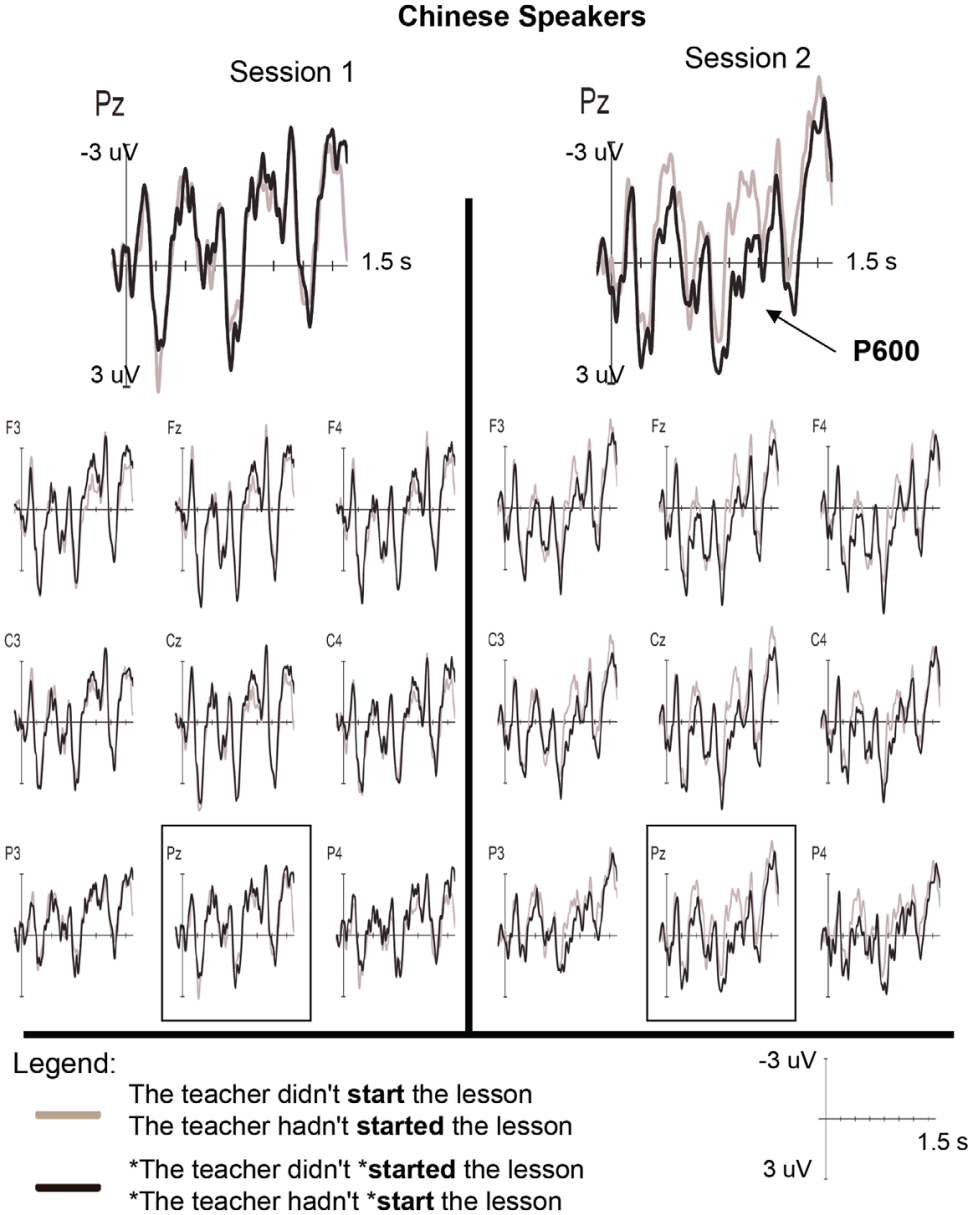
ERP data for Chinese participants. Averaged ERPs for the Chinese participants at session 1 and 2 for analysis of all trials. All time specifications are relative to the onset of the critical word. The Chinese participants exhibited a significant P600 at session 2 that was absent at session 1.

**Figure 3:**
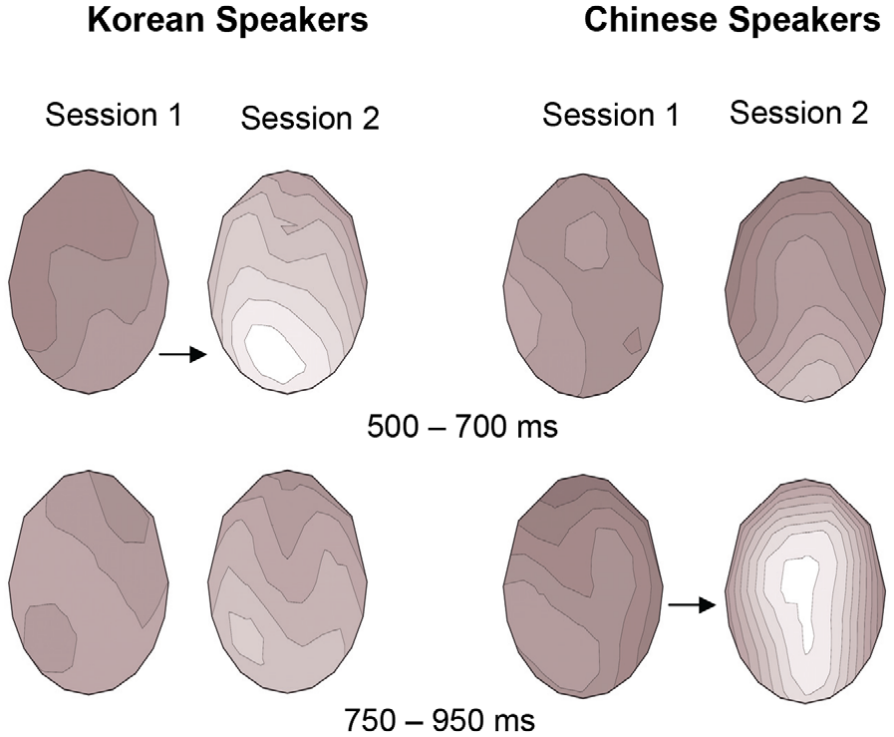
Topographical Maps. Voltage maps for the Korean and Chinese participants at session 1 and 2 in the 500–700 ms and 750–950 ms time windows. Both L1 groups exhibited significant P600 effects at session 2, although they were earlier in the Korean than the Chinese speakers.

Grand average ERP waveforms for the Korean and Chinese participants at each session are presented in **Figure 1** and **2** **,** respectively, and topographical maps are shown in **Figure 3**. Both groups of participants exhibited a positivity at session 2 in response to the tense violations that was not present at session 1. This positivity occurred earlier for the Koreans than the Chinese. Results from the global ANOVA are presented in **Table 6**. The analyses reported here are based on raw (non-scaled) ERP data. Additional analyses conducted on vector-normalized data [52] revealed a similar pattern of results. Most importantly, both the original analyses and the analyses on normalized data revealed the exact same interactions regarding differences between sessions and between L1 groups.

**Table 6:** Summary of ANOVA F-values and degrees of freedom for comparison of the correct and violation sentences in the analysis of all trials using L1 as a between-subjects variable.

		df	500–700ms	750–950ms
**A.**	Con	1, 30	>1	3.69+
	Con (mid)	1, 30	>1	4.97*
	Con×Lat	1, 30	2.08	6.85*
	Con×Hem	1, 30	1.50	5.04*
	Con×AP	2, 29	2.85*	3.75*
	Con×AP (mid)	2, 29	3.41*	2.04+
	Con×Lat×Hem	1, 30	3.75+	5.84*
	Con×Lat×AP	2, 29	>1	2.52+
	Con×Lat×L1	1, 30	>1	**5.44** *
**B.**	Sess×Con	1, 30	2.62	3.01+
	Sess×Con (mid)	1, 30	3.04+	3.80+
	Sess×Con×Lat	1, 30	7.57**	4.82*
	Sess×Con×Lat×L1	1, 30	**4.99** *	>1
	Sess×Con×Hem×L1	1, 30	**4.90** *	1.58
	Sess×Con×Lat×Hem×L1	1, 30	1.89	4.07+
	Sess×Con×Lat×Hem×AP×L1	2, 29	2.60+	**6.37** *

+ *p*≤.10.

* *p*≤.05.

** *p*≤.01.

*** *p*≤.001.

Con = Condition, AP = Anterior-Parietal, Hem = Hemisphere Sess = Session, L1 = L1-background; Mid = midline.

A. = Effects shared across sessions.

B. = Changes between sessions.

Significant group differences are highlighted in bold.

Analysis of the ERP data elicited between 500–700 ms revealed the following effects: a significant Sess×Cond×Lat interaction (*p*≤.01), which was qualified by significant Sess×Cond×Lat×L1 and Sess×Cond×Hemi×L1 interactions (*ps* <.05). Follow-up analyses that were conducted for each L1 group separately suggested a change in ERP effects between sessions for the Korean group only in this time window. Indeed, the Korean participants displayed significant Sess×Cond×Lat [*F*(1, 30) = 12.29, *p*<.005] and Sess×Cond×Hemi [*F*(1, 30) = 4.49, *p*<.05] interactions as well as a Sess×Cond interaction that approached significance [*F*(1, 15) = 2.51, *p = *.051]. While no significant effect of condition was observed at session 1, the Koreans exhibited a highly significant P600 at session 2 [Cond: *F*(1, 15) = 24.48, *p*<.001], as seen in **Figures 1** and **3**. Significant Cond×Lat [*F*(1, 15) = 10.23, *p*<.01], Cond×Hemi [*F*(1, 15) = 6.51, *p*<.05], and Cond×Lat×Hemi [*F*(1, 15) = 5.91, *p*<.05] interactions at session 2 revealed that this positivity was largest at medial right [*F*(1, 15) = 25.09, *p*<.001], medial left [*F*(1, 15) = 23.88, *p*<.001], and lateral left sites [*F*(1, 15) = 19.17, *p*≤.001]. Similarly, at midline electrodes, a significant positivity was observed at session 2 [*F*(1, 15) = 16.24, *p*≤. 001] that was not present at session 1 [*p*s >.10], resulting in a significant Sess×Cond interaction [*F*(1, 15) = 4.71, *p*<.05]. For the Chinese participants, in contrast, no significant ERP effects or change in effects between sessions was observed in this time window (*p*s >.10).

Between 750–950 ms, a number of significant interactions involving the factors Cond, Lat, Hemi and Ant-Post were observed, including a significant Sess×Cond×Lat×Hemi×Ant-Post×L1 interaction (*p*<.05; see **Table** **6**). Analyses of each group separately revealed a significant Sess×Cond interaction for the Chinese participants [*F*(1, 15) = 7.13, *p*<.05], indicating a change in ERP effects between sessions in this later time window. Specifically, a significant positivity was observed at session 2 [Con: *F*(1, 15) = 10.22, *p*<.01] that was not seen at session 1. Significant Cond×Lat [*F*(1, 15) = 8.97, *p*<.01] and Cond×Lat×Ant-Post [*F*(1, 15) = 3.61, *p*<.05] interactions in session 2 revealed that this positivity was largest at medial central [*F*(1, 15) = 11.59, *p*<.005], medial posterior [*F*(1, 15) = 13.22, *p*<.005] and lateral posterior [*F*(1, 15) = 12.55, *p*<.005] electrodes, consistent with P600 effects reported in previous studies. Similarly, at midline electrodes, a significant positivity was observed at session 2 [*F*(1, 15) = 15.77, *p*≤.001] that was not present at session 1 [*p*s >.10; Sess×Cond: *F*(1, 15) = 13.07, *p*<.005]. The only effect observed for the Chinese participants at session 1 was a Cond×Lat×Hemi interaction [*F*(1, 15) = 5.24, p<.05]; however, unlike the effects for session 2, this did not lead to a significant main effect of Cond (*p>*.10).

For the Korean participants, two interactions approached significance in this time window: Sess×Cond×Lat×Hemi [*F*(1, 15) = 3.84, *p = *.069] and Sess×Con×Lat×Hemi×Ant-Post [*F*(1, 15) = 3.44, *p = *.051]. Analysis of each session revealed no significant effects at session 1 and significant Cond×Hemi [*F*(1, 15) = 6.57, *p*<.05] and Cond×Lat×Hemi [*F*(1, 15) = 6.81, *p*<.05] interactions at session 2. However, unlike the effect exhibited by this group in the earlier time window, these interactions point to only a marginally significant positivity at lateral left electrodes [*F*(1, 15) = 3.8, *p = *.07].

### ERP Results: Analysis by L1 Groups (Correct Only Trials)

The main results from the analysis of correctly-answered trials are summarized in **Table 7** **.** Overall, these data mirror the findings reported for the analyses across all trials. Again, we confirmed the emergence of a significant positivity at session 2 for both the Korean and Chinese participants that was not present at session 1. As in the analysis of all trials, this positivity began later in the Chinese than in the Korean participants. In response-contingent analyses the Koreans’ positivity extended into the later time window.

**Table 7:** Summary of ANOVA F-values and degrees of freedom for comparison of the correct and violation sentences in the analysis of correctly answered trials only using L1 as a between-subjects variable.

		df	500–700ms	750–950ms
**A.**	Con	1, 19	>1	6.41*
	Con (mid)	1, 19	1.16	5.46*
	Con×Lat	1, 19	1.67	8.77**
	Con×AP	2, 18	2.49*	8.74***
	Con×AP (mid)	2, 19	4.51*	4.31**
	Con×Hem×AP×L1	2, 18	**3.74** *	2.25
**B.**	Sess×Con	1, 19	1.19	9.49**
	Sess×Con (mid)	1, 19	2.08	9.30**
	Sess×Con×Lat	1, 19	6.504*	6.67*
	Sess×Con×L1	1, 19	**4.66** *	>1
	Sess×Con×L1 (mid)	2, 18	3.30+	>1
	Sess×Con×Lat×L1	1, 19	>1	>1
	Sess×Con×Hem×L1	1, 19	**8.48** **	**8.53** **
	Sess×Con×Lat×Hem×L1	1, 19	2.19	3.54+
	Sess×Con×Lat×AP×L1	2, 18	2.38+	1.15
	Sess×Con×Lat×Hem×AP×L1	2, 18	>1	1.40

+ *p*≤.10.

* *p*≤.05.

** *p*≤.01.

*** *p*≤.001.

Con = Condition, AP = Anterior-Parietal, Hem = Hemisphere Sess = Session, L1 = L1-background; Mid = midline.

A. = Effects shared across sessions.

B. = Changes between sessions.

Significant group differences are highlighted in gray.

Between 500–700 ms, significant Sess×Cond×L1 and Sess×Cond×Hemi×L1 interactions were observed (**Table 7**). Follow-up analyses revealed that the Korean participants showed significant Sess×Cond [*F*(1, 10) = 5.54, *p*<.05], Sess×Cond×Lat [*F*(1, 10) = 10.21, *p*≤.01], Sess×Cond×Hemi [*F*(1, 10) = 7.43, *p*<.05] and Sess×Cond×Lat×Hemi×Ant-Post [*F*(1, 10) = 4.53, *p*<.05] interactions, reflecting the emergence of an early P600 in session 2 that was absent in session 1 (at session 1 all *p*s involving Cond >.10). The positivity in session 2 [Cond: *F*(1, 10) = 8.53, *p*<.05] was larger over medial sites [*F*(1, 10) = 12.09, *p*<.01; Cond×Lat: *F*(1, 10) = 12.17, *p*<.01] and the left hemisphere [*F*(1, 10) = 12.15, *p*<.01; Con×Hemi: *F*(1, 10) = 3.26, *p = *.10]. As in the analysis of all trials, no significant effects were observed for the Chinese participants in this time window.

Between 750–950 ms, the ANOVA for response-contingent ERP data revealed the following pattern: a main effect of Cond (*p*<.05) was qualified by significant Sess×Cond (*p*<.01) and Sess×Cond×Hemi×L1 (*p*<.01) interactions (see **Table 7**). As in the analyses of all trials, these interactions reflect a late P600 in session 2 for the Chinese participants [Cond: *F*(1,9) = 11.03, *p*<.01] that was not present at session 1 [*p*s >.10; Sess×Cond: *F*(1, 9) = 16.79, *p*<.005]. The positivity at session 2 was largest at medial [*F*(1,9) = , *p*<.01; Cond×Lat: *F*(1, 9) = 8.65, *p*<.05] and posterior [*F*(1,9) = 24.37, *p*≤.001; Cond×Ant-Post: *F*(2, 8) = 2.59, *p*<.05] electrodes of both hemispheres. For the Korean participants, we observed a pattern that differed from what analyses across all trials had suggested. That is, we found evidence that – for correct trials only – the P600 in session 2 extended into this late time window. First, there was significant main effect of condition at session 2 [*F*(1,10) = 6.11, *p*<.05]. Second, and consistent with the P600 scalp distribution in the earlier (500–700 ms) time window reported above, the late part of this positivity was also maximal over medial sites [*F*(1,10) = 6.78, *p*<.05; Cond×Lat: *F*(1,10) = 5.82, *p*<.05] and the left hemisphere [*F*(1,10) = 9.84, *p*<.05; Cond×Hemi: *F*(1,10) = 3.74, *p = *.082].

In summary, analysis of “correct trials only” largely confirmed the emergence of P600s in session 2 observed when “all trials” were analyzed. In addition, it revealed a prolonged P600 for the Koreans. Importantly, no P600 effects were observed at session 1 for either group. This suggests that the change in ERP effects observed between sessions reflects a qualitative change in processing and the emergence of neuro-cognitive processes that were not present at the beginning of the L2 course, rather than a quantitative change in the proportion of trials that participants engaged these processes.

### ERP Results: Comparison of “All Trials” vs. “Correct Only Trials”

Both the analysis of “all trials” (i.e., trials corresponding to both correct and incorrect responses) and of “correct trials only” revealed significant P600 effects at session 2 that were not present at session 1. By directly comparing these effects we can infer whether L2 learners engaged different neuro-cognitive processes for sentences that they responded to with correct versus incorrect behavioural responses (ERP effects corresponding to trials with incorrect behavioural responses were inferred by comparing the effects for “all trials” and “correct trials only” because not enough participants had a sufficient number of artifact-free trials with incorrect behavioural responses to analyze these trials on their own). If statistically similar P600 effects are observed for both analysis types, it would suggest that P600 amplitudes for a given trial are not related to L2 learners’ accuracy in detecting the violation behaviourally. Alternatively, if larger P600 amplitudes were elicited for trials that participants correctly categorized as grammatical or ungrammatical, then it would suggest a close relationship between P600 amplitudes and subsequent behavioural performance.

To compare the two analyses directly, we conducted a repeated measures ANOVA on the mean amplitude of the P600 difference wave (i.e., response to violation sentences minus correct sentences) using Analysis Type (all trials, correct only), Sess (1, 2), and time window (500–700, 750–950) as within-subjects variables and L1 background as a between-subjects variable. The 21 participants who were included in the response contingent analyses were included here. Unsurprisingly, this analysis again found the expected Sess effect [*F*(1,19) = 8.11, *p*≤.01], reflecting larger P600 effects at session 2 than session 1. Importantly, it also revealed a significant Sess×Analysis Type interaction [*F*(1,19) = 4.89, *p*<.05] and, at session 2, a highly significant main effect of Analysis Type [*F*(1,19) = 11.82, *p*<.005], pointing to a larger P600 effect for the analysis of correctly answered trials (*M* = 1.73 uV) compared to the analysis of all trials (*M* = 1.34 uV). No difference between Analysis Type was observed at session 1(*F* <1). This suggests that, at session 2, the L2 learners exhibited larger and/or less variable P600 effects for trials they responded to correctly, compared to incorrect trials. **Table 8** displays the mean amplitude of the P600 effects for each analysis type, session, time window, and group.

**Table 8:** P600 amplitude for “all trials” and “correct trials only”.

	Session 1				Session 2			
	500–700 ms		750–950 ms		500–700 ms		750–950 ms	
	All	Correct	All	Correct	All	Correct	All	Correct
**Korean**	−.08 (.83)	−.21 (1.09)	−.30 (.58)	−.30 (.80)	**1.92 (.79)**	**2.46 (.95)**	1.61 (.72)	**1.86 (.73)**
**Chinese**	.57 (.87)	.72 (1.15)	.55 (.61)	.26 (.83)	.40 (.82)	1.19 (1.0)	**2.55 (.76)**	**3.17 (7.7)**

Mean amplitude (*u*V) of the P600 difference wave (violation minus correct sentence) at electrode Pz for each group, session and time window based on “all trials” and “correct trials only”. Standard Errors are presented in parentheses. P600 effects that reached significance are highlighted in bold.

### Relationship between ERP Results and Behavioural Performance

In the analyses of the behavioural data, we found that measures of grammatical sensitivity (d-prime scores) were significantly higher after the intensive English-as-a-second-language course compared to those at the start of the course for both the Korean and Chinese speakers. ERP measures of neuro-cognitive processing were also different at session 1 and 2, with both groups displaying significant P600 effects at session 2 that were not present at session 1. Taken together, this suggests that higher levels of behavioural performance were associated with P600 effects at session 2. However, previous studies have shown that late L2 learners demonstrate considerable variability, both in terms of their behavioural performance and ERP effects (e.g., [39]). Therefore, an important question is whether an individual’s ability to discriminate grammatical from ungrammatical sentences is directly reflected by ERP measures.

**Figure 4:**
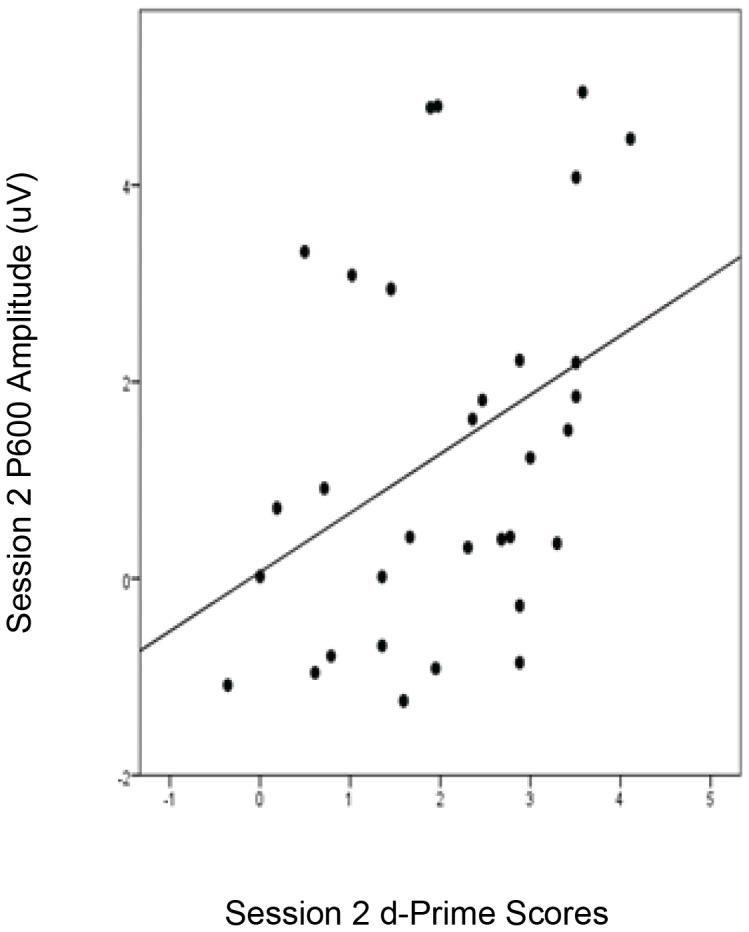
Relationship between ERP Results and Behavioural Performance. Scatter plot showing the correlation between behavioural measures of grammatical sensitivity (d-prime scores) at session 2 and P600 effects (mean amplitude of P600 difference wave at electrode Pz between 500–950 ms).

To investigate the relationship between individual learners’ behavioural performance and P600 effects, we ran bivariate correlations between the participants’ grammatical sensitivity (d-prime) scores at session 2 and the mean amplitude of session 2 P600 difference waves (violation minus correct) for “all trials” at a representative electrode (Pz) between 500–950 ms. This revealed a significant positive correlation between session 2 P600 effects and grammatical sensitivity at session 2 (*r* = .378, *p*<.05), indicating that participants with higher d-prime scores exhibited larger P600 effects. This provides further evidence for a link between higher levels of behavioural performance and larger P600 effects. This also suggests that the session 2 P600 effects reported earlier for the Korean and Chinese participants were likely driven by the participants with the highest behavioural performance. Moreover, this correlation remained significant even after controlling for P600 amplitude at session 1 (*r* = .384, *p*<.05), indicating that the relationship between brain and behavioural performance at session 2 cannot be explained by pre-existing differences in brain responses that were present at the start of the intensive course. The relationship between behavioural performance and P600 amplitude is presented as a scatter plot in **Figure 4**.

## Discussion

Four main findings emerged from this study. First, P600s effects that were absent at the start of the L2 course emerged after intensive L2 exposure for both Korean- and Chinese- L2 learners of English and, thus, regardless of L1–L2 differences. Second, generally speaking, the amplitude of the P600 effect at session 2 was largest in the L2 learners who displayed the highest levels of performance during the on-line grammaticality judgement task. These findings suggest that it is a learner's level of L2 proficiency and, in particular, proficiency with respect to the particular grammatical structures under investigation that determines access to the P600's underlying neuro-cognitive processes, rather than the grammatical structures in his/her L1. Third, a significant change in ERP effects was observed in the analysis of “all trials” as well as “correct trials only,” providing evidence for a qualitative change in the neuro-cognitive processes used by the L2 learners at the start and end of the L2 course. Finally, although all participants exhibited a P600, its onset latency was later in the Chinese compared to the Korean speakers. In what follows, we discuss how L2 proficiency influences the presence and magnitude of the P600 and how, in some cases, L1 background may influence its latency.

### The Presence and Magnitude of the P600 Reflects L2 Proficiency

Previous ERP studies of L2 grammar processing in late L2 learners have argued that a learner’s L1 background determines which neuro-cognitive mechanisms are available for L2 processing (e.g., [7], [8], [9], [10]). In particular, these studies concluded that late L2 learners will not exhibit a P600 when processing L2 morpho-syntactic structures that are expressed differently in the L1 and L2 or that are not present in the L1 at all. In contrast, the results of the present study showed that P600s can, in fact, be elicited when processing L2 grammatical features that are acquired later in life and that do not exist in the L1 (Chinese speakers) or operate differently in the L1 and L2 (Korean speakers). That P600 effects were observed in the present study after (but not before) L2 learners participated in intensive intermediate-level L2 instruction, suggests that the neuro-cognitive processes that are thought to underpin the P600 in native speakers can become available to late L2 learners as their L2 proficiency improves. Applying Osterhout et al.'s [22] interpretation of P600 effects in L2 speakers, this means that by session 2 both the Chinese and Korean speakers had “grammaticalized” the morpho-syntactic rules that differentiated the correct and violation sentences in the current experiment and had incorporated these rules into their on-line language processing system. Thus, the ability to engage these processes does not appear to be limited to L2 grammatical structures that are similar to those in the L1 and, moreover, appears to become available to L2 learners at intermediate levels of L2 proficiency (see also [23], [33]).

The emergence of P600 effects after intensive L2 instruction corresponded with overall improvement in behavioural measures of the participants’ grammatical sensitivity (as measured by d-prime scores on the grammaticality judgement task), suggesting that the processes underlying the P600 became available to L2 learners as their L2 proficiency with respect to the specific grammatical structures tested (i.e., regular past tense verbal morphology) increased. Two lines of evidence support this claim. First, there was a significant correlation between the P600 amplitude and d-prime scores at session 2, demonstrating that individuals who displayed larger P600 effects were those who displayed higher levels of behavioural performance. This corroborates the findings of Tanner et al. [26], [39] who reported significant correlations between P600 amplitude and grammatical sensitivity in their English-speaking learners of German-L2 when processing a grammatical structure (subject-verb agreement) that is present in the L1 and L2. Our results show that the same relationship between individual differences in P600 amplitude and L2 grammatical sensitivity holds for processing L2 grammatical structures that cannot be directly transferred from the L1.

Second, given the significant correlations between P600 amplitude and d-prime scores, one might also expect to find that sub-groups of participants who displayed relatively high levels of behavioural performance after the L2 course would show significant changes in ERP effects between sessions and a significant P600 at session 2, whereas those with relatively low levels of behavioural performance might show neither change nor P600 effects. In an exploratory post-hoc analysis we found just that. Using a median split of session 2 d-prime scores, subgroups of “high” and “low” performers were created. ERP effects exhibited between 500–950 ms at midline electrodes were then examined using repeated measures ANOVA.

**Figure 5:**
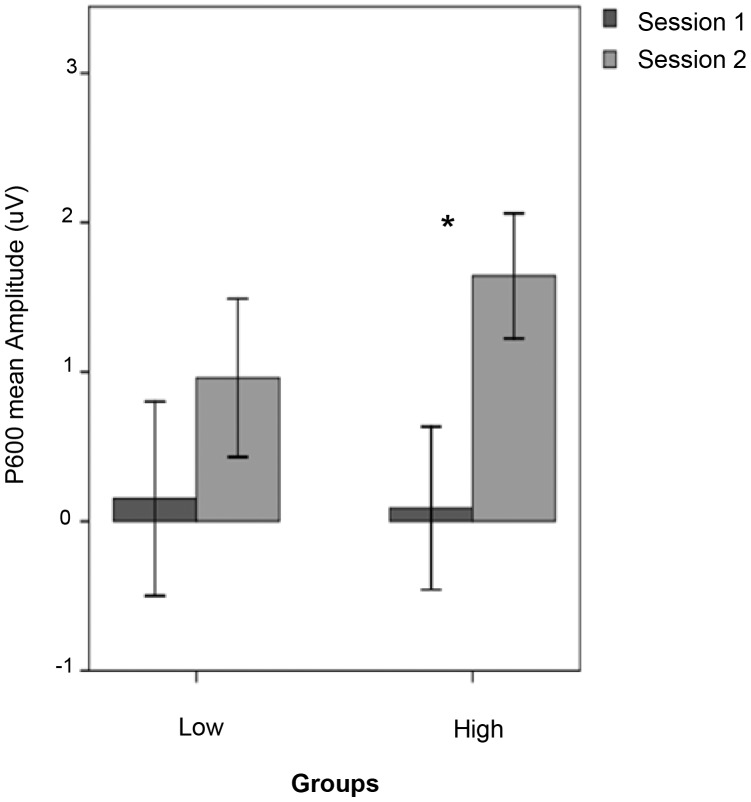
P600 Amplitude in “High” and “Low” Performance Groups. Mean amplitude of the P600 difference wave elicited at electrode Pz between 500–950 ms at session 1 and session 2. Only the “high” group showed a significant change in ERPs between sessions and a significant P600 effect at session 2. Error bars represent +/−1 Standard Error.

These results are consistent with the idea that higher levels of behavioural performance at session 2 and learning (as measured by improvements in behavioural performance from session 1 to 2) are associated with the development of the P600. The “high” performance group showed a significant change in ERP effects between sessions [*F*(1, 14) = 10.53, *p*<.01] and a highly significant P600 at session 2 [*F*(1, 14) = 9.84, *p*<.01], whereas the “low” group showed neither significant change nor session 2 P600 effects (*ps >.*10). As one would expect, this mirrors behavioural performance as well. The “high” group showed a significant improvement in their d-prime scores [*F*(1, 14) = 19.44, *p*≤.001], whereas the “low” group displayed only marginally significant improvement [*F*(1, 14) = 19.44, *p*≤.001]. No effect of L1 background was observed in any of these analyses. Together, this is evidence for a direct link between neuro-cognitive processing and the behavioural performance that is associated with this processing. The difference between the mean amplitude of P600 effects exhibited by the “high” and “low” groups at each session can be seen in the bar graphs in **Figure 5**.

In line with previous suggestions by Steinhauer and colleagues [6], the results of this study underscore how indices of proficiency with respect to the *specific structures* used in the ERP experiment (i.e., d-prime scores) may be more appropriate indicators of the neuro-cognitive mechanisms used during L2 grammar processing than general measures of L2 proficiency, as has been used in some previous studies. Previous studies of L2 morpho-syntax processing may not have observed P600s because some of the learners tested displayed low levels of structure specific proficiency, despite relatively high levels of general L2 proficiency (e.g., [9]). Indeed, in the present study, individual differences in structure-specific L2 proficiency were found over time and these corresponded to different profiles of ERP effects, even though all participants were enrolled in an intermediate level English-as-a-L2 course. In line with the findings of Osterhout and colleagues [22], [25], [26], [37]–[39], our results indicate that, although a group of L2 learners may appear to be homogeneously proficient overall, substantial differences can exist among individuals with respect to their proficiency in particular grammatical structures and, moreover, the neuro-cognitive mechanisms they use to process those structures. In addition, the results of study show that this pattern also holds for structures that differ between L1 and L2. Future research will benefit from exploring this source of individual variation further.

An important question we also sought to address is whether the emergence of P600 effects at session 2 reflected a qualitative or quantitative change in neuro-cognitive processing between sessions. Had we only conducted analysis of “all trials” (i.e., correct and incorrectly answered trials combined) we would not have been able to tease these possibilities apart. It could have been argued that P600 effects may have been exhibited at both sessions for correctly answered trials, but that these effects did not reach significance at session 1 because they were observed by the relatively large number of trials with incorrect responses. We would not have been able to rule out the possibility that the L2 learners engaged similar neuro-cognitive processes at the start and end of the intensive L2 course, but differed in how consistently they used them – a quantitative change in neuro-cognitive processing. However, this explanation cannot account for the results of the “response contingent analyses”, in which no significant P600 effects were observed at session 1, even when correctly answered trials were analyzed on their own. P600s were, however, observed at session 2. This suggests, instead, that the emergence of the P600 at session 2 reflects a qualitative change in processing and the recruitment of neuro-cognitive mechanisms that were not available to the L2 learners only 9 weeks previously. This corroborates the findings of artificial language learning experiments that report the emergence of P600 effects when processing grammatical structures that are not present in participants’ L1 after relatively short training periods, particularly when learners are immersed in the L2 environment as in the present study [24], [34].

Analyzing “all trials” was beneficial in that it allowed us to contrast our findings to those of previous ERP studies of morpho-syntactic processing that tested L2 learners with low-intermediate levels of L2 proficiency (e.g., [8, 9 experiment 2, 10, 28, [39]). Moreover, by directly comparing the results of “all trials” and “correct only trials” we could also speculate as to whether the L2 learners engaged different neuro-cognitive processes for sentences that they responded to correctly or incorrectly. Previous studies have reported significant changes in the ERP components exhibited by L2 learners even before corresponding changes in behavioural measures of language processing occurred, suggesting that ERP measures are more sensitive to learning progress than behavioral measures [48], [49]. Thus, it could have been that trials with both correct and incorrect behavioural responses elicited similar P600 effects in the present study as well. However, this comparison revealed larger P600 effects for the analysis of “correct trials only” compared to the analysis of “all trials”. Additionally, for the Korean participants, the duration of the P600 was longer for trials they responded to correctly. We can infer from this that trials that corresponded to incorrect behavioural responses either did not elicit P600 effects or the effects were smaller and less consistent than those elicited by correctly answered trials. This corroborates the results of Osterhout and Mobley [20] who found that sentences containing gender agreement violations involving personal pronouns elicited P600 effects only in a sub-set of native speaker participants who deemed the sentences to be unacceptable. This reinforces the tight link between behavioural performance and P600 effects and suggests that P600s may be a marker of morpho-syntactic processing only for trials and in participants who detect the violations as such.

### L1 Reading Strategies may Influence the Latency of the P600

Although both the Chinese and Korean speakers exhibited significant P600 effects at session 2, for the Chinese speakers this effect started approximately 250 ms later than for the Korean speakers. In studies of L2 grammar processing, delayed P600s have often been attributed to low levels of L2 proficiency (e.g., [6], [53]). For example, Rossi et al. [29] reported P600 effects that began approximately 300 ms later for low- compared to high-proficiency L2 learners in all three sentence conditions tested (subject-verb agreement, word category, and combinations of both types of violations). In the present study, however, differences in L2 proficiency are unlikely to account for the latency differences observed between the Chinese and Korean speakers because both groups displayed similar levels of behavioural performance on the grammaticality judgement task (as measured by d-prime scores), rated their English abilities as similar, and received comparable marks at the end of their intensive English-as-a-L2 course.

It is possible that the latency difference could reflect differences between the Chinese and Korean speakers in terms of their L1 grammatical knowledge, independent of L2 proficiency. Under this account, the Chinese speakers may have been slower to initiate the processes underlying the P600 because they could not transfer any relevant grammatical knowledge from their L1, whereas the Korean speakers might have been faster because they could transfer at least some L1 knowledge of verbal inflection to aid L2 sentence processing (even though the specific nature of their knowledge was different). However, this explanation has difficulty accounting for the results of Dowens et al. [23] who compared the processing of Spanish number and gender agreement violations in native English speakers who were highly proficient late L2 learners of Spanish. P600s with similar onset latencies were observed in response to violations of both number (a grammatical structure used in both English and Spanish) and gender agreement (a grammatical structure that is not used in English), although the amplitude of the P600 was smaller in response to the gender violations. Thus, it is unclear whether L1–L2 grammatical similarities can account for the delayed latency of the P600 observed here by the Chinese speakers.

Another possibility is that the latency difference can be attributed to the transfer of reading strategies from their L1, rather than grammatical knowledge. Slower and less accurate word reading by the Chinese speakers, compared to the Koreans, may have delayed subsequent morpho-syntactic analysis and led to a later P600. Behavioural studies show that Chinese speakers are less accurate English word readers than Koreans who are matched in their level of English proficiency, particularly when differentiating between words that look alike [41]. This difficulty is thought to reflect differences in how Chinese speakers read words in their L1 and L2, compared to both Korean and English native speakers. Word reading is thought to occur in a similar way in English and Korean because they are both alphabetic languages – the phonological and orthographic representations of words are activated in a cascade style and feed forward to activate the meaning of the word being read (see [40]). Chinese, in contrast, has a logographic or morphosyllabic system; written characters correspond to spoken syllables and morphemes which, in many cases, are whole words [40]. This means that orthographic processing plays a central role when reading Chinese and that it must reach a certain threshold before the corresponding phonological and semantic representations of the word can be activated [54].

Neural imaging studies report that the network of brain areas that are activated during Chinese word reading include areas that are not consistently activated by native English speakers when reading English [55]. These areas include the left middle frontal gyrus (lMFG), which Perfetti *et al.,* [30] have suggested may be involved in maintaining the orthographic form of the character in working memory while the phonological and semantic representations of the word are retrieved and integrated. Interestingly, these same areas are activated when Chinese learners of English read in English, suggesting that they use the brain network they developed for reading their L1 when reading their L2 [56]. In other words, Chinese speakers appear to rely heavily on orthographic processing during word reading in both Chinese and English, and this may make them slower and less accurate than Korean speakers at reading individual English words and, in particular, differentiating between words that look alike.

In short, when presented with critical words in the current experiment, that differ by only two letters (e.g., didn’t start vs. didn’t *started), the Chinese speakers may have been slower at differentiating between correct and violation sentences because of the way they read, rather than the way they process the sentence’s grammaticality. Gouvea, Phillips, Kazanina and Poeppel [57] have suggested that when processing morpho-syntactic violations, the onset latency of the P600 reflects the time needed to recognize and retrieve the incoming verb, access the relevant features of the noun phrase from working memory, detect a mismatch, and begin sentence reanalysis. Thus, if the system is slowed down at the word reading level, morpho-syntactic processing may also be delayed. For the Chinese speakers, reading English words as they would read Chinese characters is a less efficient strategy that may have resulted in a P600 with a delayed onset. For the Koreans, in contrast, this would not have been a problem because they are accustomed to using both phonological and orthographic information during word identification. As a result, the Koreans exhibited a P600 whose latency was similar to that observed previously in English native speakers [42]. Thus, the delayed latency of the P600 may reflect of the transfer of L1 reading strategies rather than grammatical features. This explanation may also account for the lack of a P600 before 1000 ms in previous reading studies of morpho-syntactic processing by Chinese L2 learners of English [7], [28], [31]. It may also explain why a P600 was not observed prior to 1000 ms in the high proficiency Japanese L2 learners of English reported by Ojima et al. [8], as Japanese Kana is a syllabic writing system that also relies heavily on orthographic processes during word reading [40]. Rather than exhibiting no P600 whatsoever, the L2 learners in these studies may have displayed a delayed P600 that was not evident in the 1000 ms time window that was used to analyze the waveforms. If it is indeed the transfer of L1 reading strategies that influenced the latency of the P600, rather than L1 grammatical knowledge, this would suggest that Chinese learners of English may display P600s with earlier onset latencies if they are required to listen to experimental sentences rather than read them. Comparing the latency of the P600 in Chinese speakers as they either read or listen to English sentences containing morpho-syntactic violations would elucidate this issue further.

It is important to highlight that, viewed from this perspective, differences in latency of the P600 is a not a “fundamental difference” between groups. In the current study, both the Korean and Chinese L2 learners of English displayed a P600 at the second testing session that differed primarily with respect to *when* it was exhibited. That is, group differences were quantitative rather than qualitative. This demonstrates that after intensive L2 instruction, both groups were able to apply the same neuro-cognitive mechanisms during L2 morpho-syntactic processing that are thought to be reflected by the P600 in native English speakers. Moreover, it is possible that the difference between the Chinese and Koreans in terms of the onset latency of their P600 effects would disappear if the Chinese speakers participated in intensive training that focused specifically on improving their English word reading skills (e.g., by learning how to segment the phonological information contained in printed English words). Indeed, in the present study no explicit instruction about word-level decoding strategies was provided to students during the intensive L2 course, which could account for the transfer of L1 reading strategies during L2 reading. If word-level reading training did result in a change in P600latency, it would further underscore how deployment of processes underlying the P600 are not restricted to grammatical rules acquired early in life, but rather change with learning and can be applied to new structures, even in adult learners.

### Future Research Directions

Before concluding, we would like to highlight three more potential avenues for future research. First, behavioural studies indicate that L2 learners are more likely to make errors of omission in production (i.e. omitting required inflections) than errors of commission (i.e. adding inflections where none is called for) [58]. Thus, processing of these types of errors may also engage different neuro-cognitive processes, especially at early stages of L2 proficiency. In the present study, these violation types were combined in order to avoid ERP artefacts that arise from using different word forms in the correct and violation conditions and more trials per sub-condition would have been required to investigate this systematically. Future work that tracks how processing of these types of violations change as L2 proficiency increases would provide a better understanding of how knowledge of L2 verbal inflection develops during L2 acquisition in late learners.

Second, a number of studies by Osterhout and colleagues [25], [37], [38] suggest that L2 learners may pass through an intermediate stage of neuro-cognitive processing during which they exhibit N400 effects in response to morpho-syntactic violations, before they advance in proficiency and display P600s as found in native speakers. The N400 stage has been interpreted to mean that L2 learners may initially memorize inflected words as unanalyzed “chunks” rather than decomposing them into stem and affix sequences. However, the relationship between these processes and behavioural measures of L2 performance is unclear. For example, in their study of L2 morpho-syntactic processing, Tanner et al. [39] report that high levels of grammatical sensitivity performance was associated with larger P600s and *smaller* N400s, suggesting the neuro-cognitive processes underlying the N400 effect may not be as closely linked to behavioural performance as P600 effects. However, these findings have been based primarily on analyses of ERP effects corresponding to “all trials”, irrespective of behavioural responses. Thus, it is unknown to what extent N400s are exhibited when processing sentences that participants respond to correctly or incorrectly. To this end, directly comparing the ERP effects elicited in response to “all trials” versus “correct trials only” (as in the present study) may help reveal the relationship between different kinds of neuro-cognitive processes and behavioural performance.

Finally, one of the goals of the present study was to examine the relationship between behavioural measures of L2 performance and underlying neuro-cognitive processing, as indexed by P600 effects. The results of the analyses comparing “all trials” and “correct only trials” suggested larger and more consistent P600s were elicited for trials that participants responded to correctly compared to incorrect trials. However, a limitation of the current study, as with most ERP studies of L2 morpho-syntactic processing, is that performance was assessed with a two-choice grammaticality judgement task; participants were required to respond even if they were not sure of a sentence’s grammaticality. Thus, it is likely that a portion of correct responses occurred as a result of chance, rather than the learners’ L2 grammatical knowledge. One possibility for future studies would be to assess performance on a grammaticality judgement task using a Likert-type scale (i.e., participants rate each sentence’s grammaticality using a 1–5 scale) or to ask participants to indicate their confidence in their grammaticality judgement ratings. Such an approach would decrease the likelihood that correctly-answered trials were due to chance and, therefore, provide a more sensitive measure of the relationship between behavioural performance and ERP effects. It is also possible that small ERP effects might be observed for sentences that participants rate as “somewhat ungrammatical” and larger ERP effects for sentences rated as “definitely ungrammatical”. If so, this would indicate that participants could, on some level, distinguish the sentence’s grammaticality although they were not confident in their subsequent behavioural judgements. Comparing ERPs as a function of these response types might provide sensitive information about the neuro-cognitive processes that L2 learners engage as they advance in proficiency.

### Conclusion

The results of this study reveal that qualitative neural changes can co-occur with L2 learning in adults, even when processing L2 morpho-syntactic structures that either operate differently in the L1 and L2 or that are not present in the L1 at all and, thus, cannot be directly transferred from their L1. Moreover, the findings that P600s were: (1) observed after, but not before, participating in an intensive L2 course; (2) largest in L2 learners who displayed the highest levels of grammatical sensitivity; and (3) larger in response to trials that corresponded to correct behavioural responses, strongly suggest that L2 proficiency plays a critical role in determining access to the P600’s underlying neuro-cognitive processes, rather than L1–L2 similarities.
